# Key Considerations in the Design of a Sentinel Network for Drug Use: Qualitative Findings

**DOI:** 10.21203/rs.3.rs-8165107/v1

**Published:** 2025-12-15

**Authors:** Sara Esteves Araujo Correia, Leonor Varela-Lema, Linda B. Cottler, Jasjit S. Ahluwalia, Angela J. Torres Iglesias, Mónica Perez-Rios

**Affiliations:** University of Santiago de Compostela; University of Santiago de Compostela; University of Florida; Brown University School of Public Health; University of Santiago de Compostela; University of Santiago de Compostela

**Keywords:** drug use, sentinel surveillance, qualitative research, public health

## Abstract

**Background:**

An effective surveillance program of drugs and addiction requires timely detection of emerging consumption patterns and new psychoactive substances. The time lag between conventional reporting systems and population-level perception creates a gap that can be more effectively addressed through sentinel networks.

**Methods:**

A qualitative study was conducted between February and May 2025. A total of 13 regional, national and international experts in public health, drug policy, prevention, epidemiology, and digital technologies participated in three focus groups and four in-depth interviews. Data were analyzed thematically using ATLAS.ti, with triangulation of researcher data with adherence to SRQR guidelines to ensure rigor.

**Results:**

Four categories and 13 subcategories were identified for the design of a sentinel network on drug use: (1) Definition and criteria for activating sentinel alerts; (2) Selection of key informants;(3) Management and temporality of alerts; and (4) Enabling factors for sustainability.

**Conclusions:**

A sentinel network for drug use should be multidisciplinary, flexible, and integrated into existing surveillance structures. Incorporating diverse perspectives and information, ensuring adequate professional support, and establishing mechanisms for motivation and sustainability are essential. This system could enhance early detection and serve as a model for substance use surveillance in comparable contexts.

## BACKGROUND

Surveillance of drug use and the determinants of use remains a constant challenge for public health systems. Despite advances in surveillance methods, there is a time lag between changes in consumption patterns occurring in community settings and their detection by surveillance systems. This delay often leads to late detection of emerging phenomena, such as the use of new psychoactive substances (NPS), shifts in patterns of use or changes in the profile of consumers (1).

An international review of drug surveillance systems showed that they commonly rely on consolidated data and are structured for the monitoring of defined indicators (1). However, these systems are not designed to capture signals from more informal settings such as social networks or local non-governmental agencies (NGOs), highlighting the need for complementary approaches with greater contextual sensitivity (2).

To strengthen their surveillance systems, some countries have adopted a sentinel approach based on the collection of signals from the active participation of professionals or strategic sites that serve as “key informants”. The National Drug Early Warning System (NDEWS) in the United States is an outstanding example of an integrated approach to drug surveillance. This early warning system employs multiple forms of surveillance, in which key informants from diverse fields act as a sentinel site director, referring signals to the coordinating center. Through this approach, the system enables a comprehensive characterization of substance use and facilitates the early identification of emerging drugs and their availability (3). At the European level, the European Union Drug Agency (EUDA) has also promoted sentinel surveillance through the Euro-DEN Plus program. This initiative collects information from hospital emergency departments across several European countries and integrates it with information from other indirect toxicological sources. Each country participating in this network contributes its own data, enabling the identification of emerging patterns and the activation of coordinated alerts within the framework of a shared European surveillance system (4).

Building on a previous qualitative study (5) that explored the requirements of a sentinel network on addictions from the perspective of potential key informants, the present study focuses on the strategic aspects of developing such a network. The study aims to identify the key elements necessary for the sentinel networks management and guide a proposed pilot study evaluating the proposed network design.

## METHODS

### Design

A qualitative study was carried out from a phenomenological perspective, using small focus groups and individual interviews to collect information. The presentation of the study results followed the recommendations of the “Standards for Reporting Qualitative Research” (SRQR) to ensure quality, clarity and rigor and the general standards reporting by the American Psychology Association (APA) for qualitative research in Psychology (6, 7).

### Selection and recruitment of participants

Snowball sampling was used for participant selection. A multidisciplinary group of professionals was contacted, including those engaged in health management, policy implementation, substance use surveillance networks at regional, national, and international levels, as well as professionals involved in prevention, and experts in the use of artificial intelligence in healthcare. A total of 17 participants were contacted; 13 agreed to participate. Contacts were made in January 2025. The main reason for non-participation scheduling difficulties. The participants were contacted by e-mail to explain the study. The characteristics of the participants are described in Table 1.

### Data collection

The collection of information was carried out between February and May 2025 through three focus groups and four in-depth interviews. The first focus group was held in person at the University of Santiago de Compostela and included public health professionals with experience in drug use prevention, treatment, and management. The other two focus groups and the four individual interviews were conducted online via the Microsoft TEAMS platform. The sessions were conducted in Spanish, English or Portuguese, depending on the participant’s native language. The research team developed a moderator’s guide to orient the focus groups (Annex I) and a structured version adapted for the in-depth interviews. Each participant took part in either a focus group or an individual interview, but not both. The duration of both the focus groups and the interviews ranged from 55 to 90 minutes. All sessions were moderated by one member of the research team (SEAC), with support of an additional research team member as an observer (LV).

After informed consent was signed and permission granted for recording, the sessions were recorded. Data collection continued until discourse saturation.

### Data analysis

The analysis was carried out based on an exhaustive reading of the audio transcripts of the meetings, applying a reflective thematic analysis approach, according to the methodology proposed by Braun and Clarke (8). This process involved data familiarization, coding, theme review and definition, and report preparation. Inductive and open coding was used, which allowed the structuring of the transcriptions into categories and subcategories. The analysis was assisted by Microsoft TEAMS and ATLAS.ti 25 digital tools. A double triangulation strategy was applied, contrasting the information provided by the participants with the inferences made by the observers.

Each participant was assigned an alphanumeric code (P1 to P13), to preserve anonymity and facilitate the tracking of verbatims. The codes do not reflect the type of participation (focus group or individual interview) or the professional profile, although this information was considered during the analysis. Participants P1 to P6 correspond to regional professional profiles, P7–P8 correspond to national professional profiles, and P9–P13 represent perspectives based on international profiles.

### Ethical considerations

The Research Ethics Committee of Santiago de Compostela-Lugo deemed the research project was exempt from formal review.

## RESULTS

### Categories and subcategories

Four main categories and 13 subcategories were identified. The main categories were: 1) Definition and Criteria for Sentinel Alert Activation, 2) Selection of Key Informants, 3) Alert Management, Analysis and Timing, and 4) Enablers for Success and Sustainability. Each of these categories is broken down into specific subcategories, as shown in Table 2.

The results of the study are presented below in the five main categories identified.

### Category 1. Definition and Criteria for Activating Sentinel Alerts

The concept of “sentinel alert” generated doubts in both the focus groups and the interviews. Participants were not provided with formal definitions and thus drew on their own professional experience to respond. Overall, they agreed that, at the local level, gets reported are signals, that is, suspicions or changes related to consumption patterns or social determinants of drug use. However, the distinction between a signal and an alert, and the point at which a signal should be escalated to an alert, remained unclear. It is emphasized that not all signals justified an immediate alert, and it may be necessary to observe their repeated existence in different sources or places prior to their activation. Nonetheless, factors such as the novelty of the substance or the magnitude of its observed impact may justify accelerating its classification as an alert, even in the presence of limited evidence.

In order to distinguish between normal variations and epidemiologically significant phenomena, it was proposed to establish activation thresholds. These thresholds could be defined based on historical data on recurring patterns of signals observed across multiple sources.

With respect to the sources of information, the combined use of quantitative and qualitative data was proposed. While some advocate for the strength of the former, others stressed the limitations of accessibility of such data and emphasize the contextual value of the latter. Triangulation of data, both direct (professional testimonies) and indirect (data from death certificates or sewage analysis), was considered key to strengthening the validity of alerts and facilitating their prioritization.

To improve the collection and comparison of signals, it was suggested to develop common epidemiological indicators, applicable to different informant profiles. However, some participants with previous experience in other sentinel networks pointed out the difficulty in applying the same set of indicators to heterogeneous informants (Table 3).

### Category 2. Selection of Key Informants

Participants emphasized the importance of integrating diverse professional profiles. Priority was given to the inclusion of public-sector professionals from the health and social care fields (physicians, nurses, pharmacists, social workers, and addiction treatment staff), the education sector (teachers, school counselors, and representatives of parent–teacher associations), and the community sector (NGOs, neighborhood associations, sports clubs). Participants also highlighted the potential value of incorporating less common profiles into sentinel networks, such as forensic personnel, law enforcement, or urban cleaning workers, given their early or indirect contact with drug-related phenomena.

There was disagreement about the role of people who use drugs as informants. Some consider their representation through social support networks to be sufficient or argue that the information they can provide can already be collected through other channels (such as care services or existing registries), while others defend their direct participation, stating that their lived experience provides valuable information that cannot be obtained any other way.

The potential inclusion of the private healthcare system was also mentioned because consumption patterns may differ from those detected in the public setting. However, it was emphasized that the relevance of this sector may vary between different regions, suggesting the need to adapt the design of the sentinel network to the institutional particularities of each territory (Table 4).

Given the potential number of key informants, it was proposed to incorporate the figure of a community-level sentinel referent. This person would act as a coordinator between multiple informants, identifying information that deserves to be reported at the regional level. It was suggested that this sentinel referent should ideally receive basic training in signal identification, analysis, and interpretation to filter out irrelevant signals and prevent overloading the surveillance system.

### Category 3. Management and Timing of Alerts

To ensure an organized and efficient management and flow of information, several participants proposed the creation of a regional advisory group (AG), composed of representatives from different sectors (such as public health, mental health, and epidemiology), with technical capacity and legitimacy for decision-making. This AG would act as an intermediate in the management of alerts. It would be in charge of receiving, analyzing and assessing alerts from the local referents. It was emphasized that its functioning should be agile from the operational point of view, avoiding bureaucratic processes that delay the response to emerging alerts.

The use of Artificial Intelligence (AI) in data management emerged as a recurring theme across all focus groups and interviews. Participants valued AI as a tool to support the detection of emerging signals from open digital sources, such as social networks or online market platforms, which can indirectly complement signals collected by professionals in the field. AI could also facilitate the rapid and efficient identification of emerging patterns or anomalies. However, several participants stressed that AI should be understood as a complementary tool rather than a substitute, since human interpretation remains essential to validate the relevance of signals. From an operational perspective, training committee members in the basic use of AI was considered more practical than relying exclusively on external technical experts. The international experience of N-DEWS (USA) was cited as an example, where AI has proven useful but limited to some platforms as it faces legal and technical limitations, particularly in contexts involving sensitive information.

To improve the efficiency of the sentinel system, several experts suggesting establishing a fixed periodicity, such as weekly or monthly cycles, for the review and communication of alerts. However, it is noted that, in the context of a sentinel network, a rigid periodicity could be counterproductive (Table 5).

### Category 4: Enablers for success and sustainability

Participants emphasized the need to provide prior training for sentinel reporters and key informants, focusing on the identification of relevant signals and the efficient use of technological tools. They also highlighted the value of periodic meetings (preferably some face-to-face sessions) to strengthen commitment and foster a sense of belonging to a collaborative network, as well as to facilitate the presentation and sharing of relevant information among participants.

The participants also highlighted the need to provide feedback to key informants to maintain commitment and enthusiasm for the network. They believed that additional incentives are needed to maintain informer enrollment, given the significant workload involved. Among the proposals are career development, time off and financial compensation structured around ongoing contributions, with clear expectations for participation and follow-through. Finally, a clear distribution of responsibilities within the network, coordinating body and AG is recommended to ensure operational sustainability and avoid work overload (Table 6).

## DISCUSSION

This qualitative study has identified key elements for the implementation of a national sentinel network for monitoring the determinants of drug use. The importance of having an operational structure that allows information to be processed and prioritized, as well as integrating multiple sources of information and key informants is emphasized.

One of the main challenges identified concerned the difficulty in clearly distinguishing between a signal and an alert. These concepts are often conflated in the literature, and this lack of clarity can complicate both the design and functionality of a sentinel network. For instance, some models distinguish between content (signal) and channel (alert), while others define an alert as the aggregation of multiple signals (9). Others like the French sentinel network, *Addictovigilance*, define a signal as an individual information related to an isolated event or case and, an alert, to a structured response once combined signals are interpreted as representing a significant risk (10). This is consistent with the findings of our study, where participants agreed that what is initially reported are signals, i.e. indications, suspicions or changes related to consumption patterns or social determinants. Therefore, for our network, we will adopt a similar approach to that used in *Addictovigilance*. Nevertheless, drawing a strict dividing line between the two terms remains difficult, as a particularly relevant signal may, on its own, constitute an alert. This highlights that a universally accepted distinction is lacking and the need for greater conceptual clarity in future work.

Participants in our study emphasized the importance of establishing a broad and diverse network of key informants. They noted that limiting informant identification to health, educational, or community settings may be insufficient to capture the complexity of substance use. Accordingly, they highlighted the value of including other professional profiles, such as forensic experts or municipal cleaning staff, who may have early or indirect contact with emerging drug-related phenomena. The latter may serve as privileged observers, as they are often in contact with waste associated with drug use. Previous studies support this perspective. Wenger et al. documented the presence of syringes and other consumption debris on the streets of San Francisco (11), and Thompson et al. focused on syringe accidents among janitorial workers in Mexico City (12).

In our study, some participants suggested that people with lived experience could be included as key informants within the network. Consistent with our previous research (5), the suitability of this informant profile generated some discussion. Several participants defended their value as a direct source for detecting emerging practices, patterns of use or substances that are not officially registered. However, others expressed reservations about the reliability of the information provided, suggesting that these data could be obtained through indirect channels, such as services for drug addicts or toxicological analyses. In a recent study, the NDEWS network reported that integrating biological, clinical, and digital data enhances reliability while ensuring continued input from individuals with lived experience (13).

The need to broaden the scope of the network, both in terms of profiles and settings, was also an issue raised in this study and the previous one (5). In the current study several participants suggested that cross-referencing clinical, community, qualitative and structured data could be key to avoiding fragmented readings, an approach already applied in Addictovigilance and in the NDEWS weekly briefing (3, 10). A commentary published in Drug and Alcohol Review (14), warns that relying on a single source to analyze trends in substance use can lead to fragmented or misleading interpretations.

Several participants identified the use of AI as a tool for signal management and analysis within a sentinel network. An example is provided by the NDEWS network, where AI is used for digital surveillance in forums and social networks related to substance use. A study by Barenholtz et al. demonstrated that mentions of new psychoactive substances on Reddit preceded, in most cases, spikes in intoxications subsequently recorded in forensic databases and poison centers (15). However, this technique is an adjunct to other methods and not the only method.

Regarding the routes of signal transmission, participants debated between using a digital platform or using simpler formats, such as email. While a digital platform was seen as allowing for greater systematization, email was still considered an accessible and effective tool, particularly in contexts with limited technological infrastructure. The experience of the ESSENCE system in the United States, a sentinel network for communicable diseases, demonstrates that digital tools can be successfully combined with traditional channels, enabling hybrid models that adapt to different user profiles and resource levels (16). However, infectious diseases are quite different than emerging drug epidemics.

Throughout the analysis, several cross-cutting barriers emerged which, although not always explicitly addressed by participants, reflect potential practical limitations that could affect the network’s functionality. These included concerns about data protection and confidentiality in digital environments, the cost and feasibility of developing proprietary platforms, as well as the difficulty of integrating this network into existing institutional structures. Another key challenge identified was the difficulty of maintaining sustained engagement of professionals, especially in care settings with excessive demand. These limitations are not unique to our context or to the field of drug use. International studies in the field of public health surveillance have pointed to similar challenges, highlighting the need for institutional support, standardized regulatory frameworks and incentives to promote participation and sustain long-term commitment (17). Among the possible incentives, the role of financial compensation was especially debated. According to the results of a recent review, monetary incentives can significantly increase the participation of professionals in notification systems (18). Some participants raised ethical concerns about paying for information, arguing that participation should be based on professional commitment and public health goals. In this line. However, some studies warn that, once the financial incentive has been withdrawn, participation may decrease in the absence of other forms of recognition (19). Based on the results, we believe that a sentinel network should prioritize long-term engagement and shared accountability, rather than relying on financial incentives tied to individual reports.

This study presents some limitations. First, given the limited number of participants, it is possible that not all perspectives are captured, although it should be noted that the purpose of the study was to explore the experiences and perceptions in depth from different key positions in the field of substance use surveillance; hence the small number of participants. However, the participation of professionals at the national level with experience in drug policy was more limited due to organizational constraints and partly because some potential participants were directly involved in Spanish national surveillance program, which also funded this project. Their involvement in the national plan was perceived as a potential conflict of interest, limiting their participation.

Despite this limitations, this study has many strengths. It offers an innovative perspective by involving key institutional stakeholders in the areas of prevention, management, health care, and public health at regional, national and international levels. In addition, the inclusion of international experts and professionals with experience in technologies applied to public health offers a comparative dimension that has not been explored to date. The methodology based on individual interviews and focus groups made it possible to contrast visions from different levels of the system, generating a space conducive to individual expression, the identification of key points and collective debate on implementation in real contexts.

## CONCLUSIONS

We believe that the results provide a solid and comprehensive foundation for advancing the design of a surveillance sentinel model responsive to the evolving realities of substance use. It will be essential to consolidate the proposed model and implement a pilot study to assess its applicability in different contexts and strengthen its institutional integration with the aim of building a robust operational sentinel network aligned with the real needs of the Spanish public health system.

## Supplementary Files

This is a list of supplementary files associated with this preprint. Click to download.

- Annex1InterviewsandFocusGroupsGuide.docx

## Figures and Tables

**Table 1:** Characteristics of the study participants

Professional Category	Sex (M/F)	Professional fields
Regional experts in drug use and health management (P1–P4)	1/3	Public health and addictive behaviors - Regional health administration
Professionals from the clinical and technological field (P5–P6)	1/1	Clinical and academic settings
National experts in addiction prevention and addiction policies (P7–P8)	0/2	Academic institutions and national public administrations in the field of drugs
Professionals in international surveillance networks (P9–P13)	3/2	Epidemiological surveillance

**Table 2:** Key Aspects in the design and implementation of an Drug Surveillance Network

**Definition and Criteria for the Activation of Sentinel Alerts** Difference between signal and sentinel alert Alert activation threshold Data typology and triangulation Epidemiological indicators **Selection of Key Informants** Key informant profiles Non-classical informant profiles Local sentinel referents **Management, analysis and temporality of alerts** Alert management Use of Artificial Intelligence Temporal aspects **Enablers for Success and Sustainability** Training and capacity building Incentives for informants Task distribution

**Table 3:** Verbatims of Category 1: Definition and Criteria for Activation of Sentinel Alerts

Category	Subcategory	Verbatims
**Sentinel Alert Definition and Activation Criteria**	Difference between signal and alert	*“Usually, a signal is like something unusual is happening. So, it’s like an initial prompt (…) then when we validate that it’s being seen multiple times in different cities, then it becomes an alert…” P10* *“An alert, a sign, is a piece of information that we receive from one of our expert networks or quantitative or qualitative data, which shows a new trend that is not the usual trend in our data.” P9*
	Alert activation threshold	*“The threshold will depend on the context. (…) I think novelty plays a big role in determining how quickly a signal becomes an alert.” P10* *“I would understand that it would be a variation on previous trends, with a defined amount in accordance with these parameters, i.e., you can talk about a 20% increase over what is expected.” P8*
	Data typology and triangulation	*“(…) if you suddenly start to see spikes in overdoses in a certain location, that can be kind of the first sign that something new is happening, even before it’s in any official report.” P12* *“Sometimes it is only Reddit because sometimes there isn’t anything else out there*. *But we try to triangulate as much as we can.” P12*
	Epidemiological indicators	*“Defining indicators (…) this social perception indicator could also be identified in multiple sources that can be repeated” P1* *“we were hoping that we would have a set of indicators that everyone across the network would be able to provide. But (…) not everyone has the access to the same kinds of data, and so at that point we realized that we had to pivot and look at other things which I think has really been beneficial in the long run because we get this different kind of more qualitative more boots on the ground data.”P13*

**Table 4:** Verbatims of Category 2: Key Informant Selection

Category	Subcategory	Verbatims
**Key Informant Selection**	Key informant profiles	*“Informants can be in Drug Dependency Care Units, in* *prevention units, in institutes and schools, in emergency rooms and health centers, community pharmacies, neighborhood associations, sports associations”* P4 *“People who use drugs are extremely stigmatized, so their voices may be overlooked or not heard. (…) I think their perspectives are important.” P11* *“They can tell you: ‘here there is no heroin or cocaine’ … or who knows what other things. Yes, however, we know this by other means, not by asking them directly.”* P2
	Non-classical informant profiles	*“I would also include people who work in the field in risk reduction NGOs, even if they are not doctors, and someone from the forensic or police field” P9*. *“The street sweepers tell them how many syringes they have picked up from the ground(…) the silver paper that is picked up on the street, that the people sweeping can see, as an indicator, is different.” P8* *“I would not rule out the private side as well. Private clinics have very interesting information on consumption of certain substances that sometimes is not seen in the public network (…) There are certain consumptions that are seen first in the private sector because they have less stigma, or simply because they are of people with more resources. That can give us different signals.” P8*
	Local sentinel referents	*“They have the vision of the street, which is the important thing. But you need someone who knows how to translate that (…) local referents (…) who are trained and know whether they have to report on what they hear.” P7*

**Table 5:** Verbatims of Category 3: Alert Management and Coordination Responsibility

Category	Subcategory	Verbatims
**Alert management, analysis and timing**	Alert management	*“I believe that a sentinel committee at the regional level would be fundamental to filter and assess the signals before sending them to the national level. This would avoid the saturation of irrelevant information and ensure that what reaches the Observatory is really important (…) it could be composed of representatives from different sectors: public health, prevention, care, even social services.(…) The important thing is that this committee has the capacity to analyze, but also the legitimacy to say: this signal we’ll raise it, this one remains under observation. If there is no such technical filter, the system breaks down.” P9*
	Use of Artificial Intelligence	*“The complicated thing is not to generate models, but to interpret the variables that may be there to do so.”* P6 *“Artificial Intelligence can help us find signals that otherwise we would not see, but we are still learning how to apply it well in this field (…) the problem is that in Europe we have to work with 30 languages. We can’t make a tool that only works in one (…)” P9* *“*(…) *would also be more challenging from sort of a regulatory perspective is we looked at monitoring forums on the dark web. They in general, the people who run those forums make them very unmonitorable anyway.” P12*
	Temporal Aspects	*“I think the most useful thing has been to set a regular time period that we report whatever is the most concerning thing.” P12* *“I really do believe in weekly dissemination because we are an early warning network. That information sometimes gets old even after like a month a month (…) If we wait, the information will eventually become redundant” P10*

**Table 6:** Verbatims of Category 4: Enablers for success and sustainability

Category	Subcategory	Verbatims
**Enablers for success and sustainability**	Training capacity building	*“The simplest thing, what can be useful and can be maintained over time, is first to train them well on how to notify, what aspects they have to investigate (…)*. P4 *“There has to be training, provide a lot of training and practical training (…) That can be very stimulating for people who often do not have access to these things, people who are at the local level and do not have access.” P7* *“Maybe it would be a matter regular monthly meetings of different regional centers, coupled with a relatively simple questionnaire system (…) and with those combinations detect if new things appear.” P8* *“(…) And especially if people are passionate and do feel the sense of urgency to really connect with the network and share information.” (…)the first meeting of each month is an ideation meeting and we have discussion with our Sentinel site directors as well as other people from the larger network (…)” P11*
	Incentives for informants	*“Take care of the person who provides the information, give it back to them well and establish good links. That is also an incentive (…) Visibility is important: that they present what they have done in periodic meetings. That is basic for people.” P7* *“Many times warning systems are launched and people leave. Burnout generates demotivation (…) By giving them career points, rest hours or economic remuneration. Otherwise, people don’t stick it out.” P5*
		*“I would put in incentives, that is, the economic part I already understand that it is going to be very complicated, but if someone is going to dedicate themselves to doing this type of thing yes, from doctoral theses to working career, to professional career, level increases or some type of incentive that the public administration allows them.” P8*
	Task distribution	*“It is a lot of work to put together information weekly, and this is where this network comes into play because we do rely on people to send us in their content” P10* *“If you have people who can do these individual parts, it makes the briefing easier to do as a whole (…).” P11*

## Abbreviations

AG: advisory group
AI: artificial intelligence
APA: American Psychology Association
EUDA: European Union Drug Agency
NDEWS: National Drug Early Warning System
NGO: non-governmental agencies
NPS: new psychoactive substances
SRQR: Standards for Reporting Qualitative Research
